# Feasibility of Laparoscopic Closed Cystectomy for Hepatic Hydatid Cyst in Segments VI, VII, and VIII

**DOI:** 10.7759/cureus.13957

**Published:** 2021-03-17

**Authors:** Oseen Shaikh, Uday Kumbhar, Sandeep Bhattarai, Suresh Chilaka, Nikhil Reddy, Muhamed Tajudeen

**Affiliations:** 1 Surgery, Jawaharlal Institute of Postgraduate Medical Education and Research, Puducherry, IND

**Keywords:** laparoscopy, hepatic hydatid cyst, liver segments

## Abstract

Background

Laparoscopic closed cystectomy of the hepatic hydatid cyst (HHC) is increasingly being performed as it has improved postoperative recovery and reduced morbidity. However, laparoscopic closed cystectomy of HHC is difficult when located in segments VI, VII, and VIII. This study aimed to assess the laparoscopic closed cystectomy feasibility of the HHC when cysts are located at the difficult access site.

Methodology

Seven patients out of 13 patients of HHC treated laparoscopically in the surgery department from 2014 to 2018 were included. These patients had cysts located in segments VI, VII, and VIII of the liver. All patients received perioperative albendazole, underwent ultrasonography (USG) and contrast-enhanced computed tomography for diagnosis. We noted the demographic character of all the patients, cyst’s location, cyst size, type of the cyst, mean operative time, intraoperative and postoperative complications, duration of the hospital stay, and recurrence of the cyst.

Results

All patients underwent laparoscopic closed cystectomy of HHC. One patient had a conversion to open procedure, and one patient had an additional thoracoscopic approach added. The mean operative time was 191.86 minutes. There were no intraoperative complications. One patient had developed a surgical site infection, and three had a minor bile leak postoperatively. The hospital stay’s mean duration was four days, and there was no recurrence in the 21 months follow-up.

Conclusion

The laparoscopic closed cystectomy of HHC located at segments VI, VII, and VIII is feasible, safe, and cost-effective. A thorough preoperative evaluation, preparation, and radiological planning of the procedure should be done.

## Introduction

Hydatid disease is caused by the parasite *Echinococcus*. Hydatid disease is common in Asian countries. Although any part of the body can be affected, the liver is the most frequent organ involved. Symptoms of hepatic hydatid cyst (HHC) develop due to the adjacent structures’ compression, from the cyst’s rupture into the bile duct, pleural space, or peritoneal cavity. Patients may develop jaundice, cholangitis, or septicemia if there is an intraperitoneal rupture [1].

Treatment of HHC involves medical, percutaneous, and surgical methods. Surgical treatments can be open methods or minimally invasive procedures. Percutaneous approaches include percutaneous aspiration-injection-reaspiration (PAIR) and its modifications. Surgical excision of the cyst is the definitive management with open surgical techniques in use at various centers. Minimally invasive surgical approaches include laparoscopic approaches. Recently there are many reports published on the laparoscopic management of the HHC. However, there is always the fear of anaphylactic shock due to the cyst contents’ spillage during the procedure [2]. Early reported laparoscopic treatment of HHC was confined to simple drainage; however, more advanced laparoscopic methods are now possible, including pericystectomy and hepatic resection.

Laparoscopic closed cystectomy of the HHC is a promising approach with minimum morbidity and mortality. However, the anatomical location of the cysts plays a significant role in the laparoscopic treatment of HHC. The lesions located anteriorly, inferiorly, or on the right lateral aspect of the liver and superficial ones are easy to deal with laparoscopically. However, areas such as posterior and posterosuperior aspects of the liver are relatively critical and inaccessible areas, precluding their easy access laparoscopically. According to Couinaud’s liver segments, segments VI, VII, and VIII are challenging to access laparoscopically and often need conversion to open access like laparotomy or even thoraco-laparotomy, which increases morbidity and mortality. Here we present our experience of laparoscopic closed cystectomy of HHC located in the segments VI, VII, and VIII, with possible tips to deal with such cases.

## Materials and methods

A retrospective study included 13 patients of HHC treated laparoscopically in the department of general surgery over five years, from 2014 to 2018. Among them, seven cases of HHC located in segments VI, VII, and VIII were included in the study. All these seven patients underwent laparoscopic closed cystectomy successfully. This study intends to describe the feasibility of these seven patients’ laparoscopic treatments having posteriorly or posterosuperior located HHC. All the patients were symptomatic (Table 1).

**Table 1:** Symptomatology of hepatic hydatid cyst in seven patients.

Symptoms	Number of patients
Abdominal pain	6
Dyspepsia	1
Nausea	2
Abdominal mass	3

Out of seven, five patients were females, and two were males. Apart from biochemical investigations, the diagnosis was made on the ultrasonography (USG) of the abdomen. Confirmation of the diagnosis and segmental localization was done using the contrast-enhanced computed tomography (CECT) scan of the abdomen. Preoperatively all patients had received albendazole 15 mg/kg/day for three weeks and continued for one month postoperatively.

All the patients were operated on under thoracic epidural and general inhalational anesthesia with endotracheal intubation. The patient’s position was supine with the head high and right side up. The monitor trolley was at the right shoulder. The operating surgeon and the camera assistant were on the left side, whereas the scrub nurse was on the patient’s right side.

**Figure 1:**
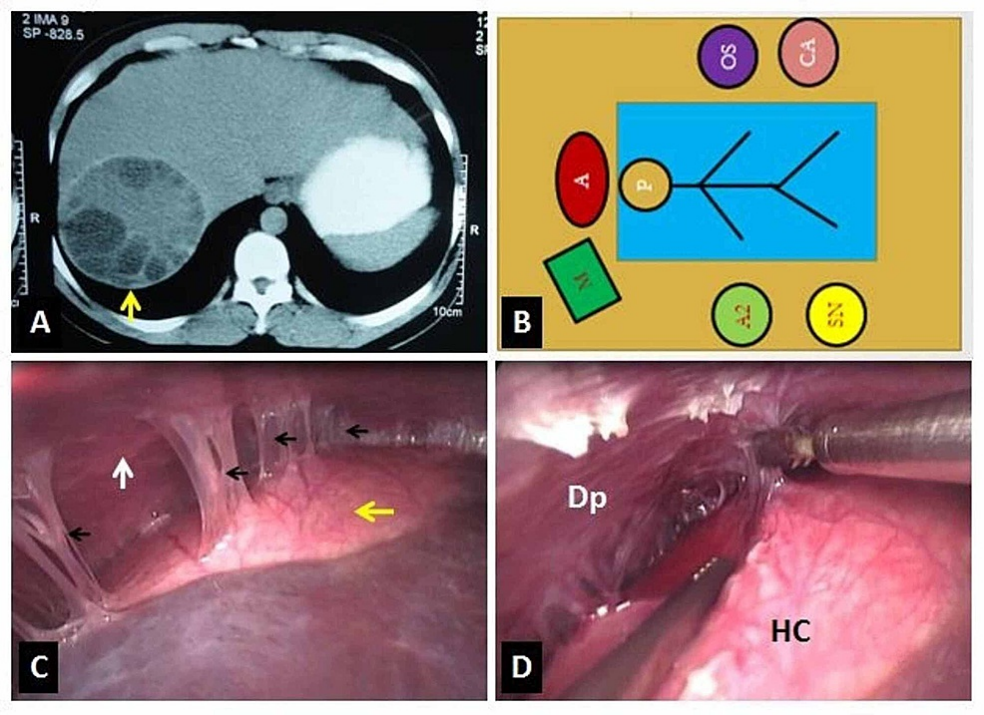
Image showing; (A) Contrast-enhanced computed tomography of abdomen showing the segmental location of the hepatic hydatid cyst (arrow), (B) Operation theatre layout (A: Anesthetist, P: Patient, OS: Operating surgeon, CA: Camera assistant, A2: Second assistant, SN: Staff nurse, and M: Monitor trolley), (C) Intraoperative laparoscopic view showing the location of the hydatid cyst (yellow arrow) and the adhesions (black arrows) between the cyst and the diaphragm (white arrow) and (D) Adhesiolysis (Dp: Diaphragm, HC: Hydatid cyst).

Pneumoperitoneum was created by an open technique using a 10 mm trocar placed 4 cm to 5 cm above the umbilicus in the midline. Laparoscopy was done to confirm the diagnosis and the position of the cyst. Another 10 mm trocar in the epigastric region 2 cm to 3 cm below xiphisternum, one 10 mm trocar at the right subcostal region in the mid-clavicular line, and one 5 mm trocar in the anterior axillary line were placed. A standard laparoscopy set consisting of a 10 mm 30 degrees telescope and a harmonic scalpel was used in all cases. In all seven cases, the cyst’s location was the posterior and posterosuperior aspect of the right lobe of the liver. The cyst had dense adhesions with the diaphragm. Adhesiolysis was done using bipolar diathermy/ harmonic shear (Figure 1).

In-house-made endo bag put intraperitoneally through 10 mm trocar. Gauze pieces soaked in 10% sodium chloride were placed around the cyst. Veress needle was passed percutaneously under laparoscopic guidance into the cyst, and 100 to 120 ml cyst fluid was aspirated. A mixture of 10% sodium chloride and 10% povidone-iodine solution was instilled into the cyst cavity through a Veress needle as a scolicidal agent and was kept for 15 minutes. The spillage of the cyst fluid was prevented using controlled suction around the Veress needle puncture site. Then, the Veress needle was replaced by a 10 mm trocar placed directly into the cyst by the Veress needle side. Possible cyst contents were aspirated through the 10 mm suction cannula passed into the cyst cavity through the 10 mm trocar. Once maximum content was extracted, the 10 mm trocar was removed, and the opening in the cyst wall was widened using harmonic shear.

**Figure 2:**
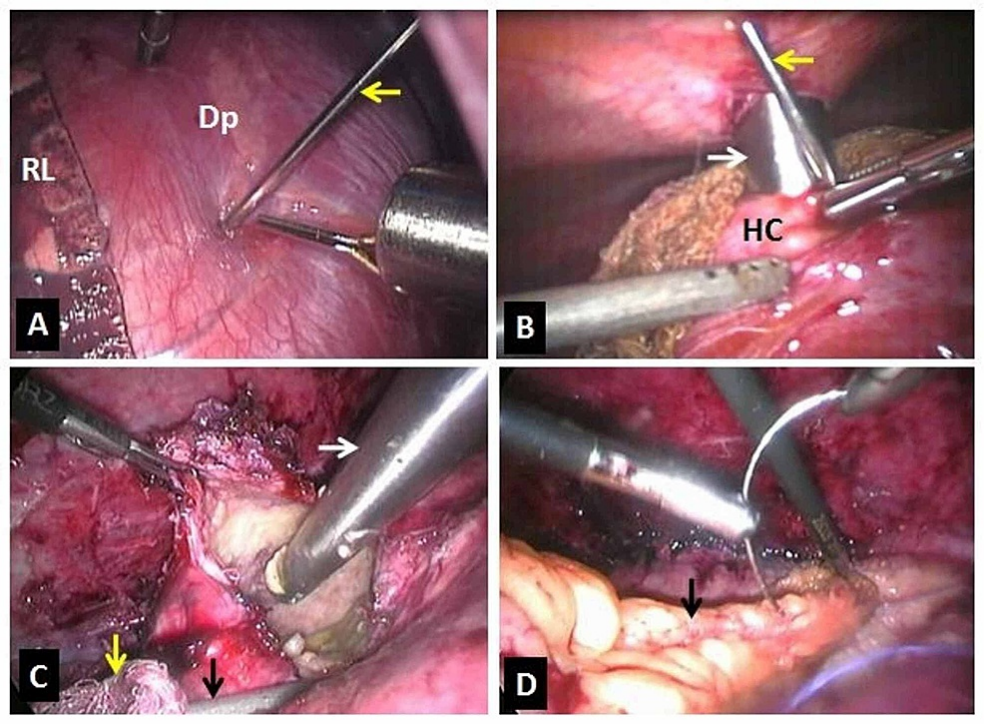
Image showing; (A) Thoracoscopic transdiaphragmatic cyst puncture with aspiration using Veress needle (arrow), and installation of the scolicidal agent in one patient (Dp: Diaphragm, RL: Right lung), (B) Placement of the 10 mm trocar (white arrow) by the side of Veress needle (yellow arrow). (HC: Hydatid cyst), (C) Opened hydatid cyst and direct aspiration of the contents using 10 mm suction cannula (white arrow). Prevention of the spillage using another suction cannula (black arrow) outside the cyst and scolicidal soaked gauze pieces (yellow arrow), and (D) Residual cyst cavity filled with omentum (black arrow).

Further, direct aspiration of the contents was done. The thick content, which was impossible to suck, was collected into the endo bag. The germinal layer was scraped using a gauze piece. The cyst wall pieces, the cyst contents, and the gauze pieces were collected in the endo bag. Proper hemostasis was achieved. The cyst’s cavity was filled with omentum, and it was fixed with the cyst wall using polyglactin suture by intracorporeal knotting (Figure 2).

The tube drain was kept in the cavity. The endo bag, along with its contents, was removed through a 10 mm supra-umbilical port after widening it. Pneumoperitoneum was deflated after the removal of all trocars under vision. All port sites were closed in layers. Patients were followed every month up to three months postoperatively and then every six months for three subsequent visits.

## Results

In the present study, out of 13 patients of HHC, seven patients had a cyst in segments VI, VII, and VIII. Five patients were female (70%), whereas two were male (20%). Among seven cases, six had solitary cysts, whereas one had multiple (three) cysts. In five patients, a closed cystectomy of a hydatid cyst was performed laparoscopically. One patient required thoracoscopic assistance in addition to laparoscopy. One patient required open conversion as there were multiple cysts, two of which were deep-seated (Table 2).

**Table 2:** Demographic, imaging, and intraoperative details of the patients with hepatic hydatid cysts confined to segments VI, VII, and VIII. CECT: Contrast-enhanced computed tomography.

Number	Age (Years)	Sex	Gharbi Type	Location of Cyst in Liver on CECT	Number of Cysts	Surgical Conversion Approach	Operative Time (Minutes)	Intraoperative Complications	Postoperative Complications
1	43	Female	2	Segments VI and VII	Single	Nil	170	Nil	Nil
2	36	Male	3	Segments VI and VII	Single	Nil	185	Nil	Nil
3	41	Female	3	Segments VI and VII	Single	Nil	168	Nil	Minor bile leak
4	35	Female	3	Segments VI and VII	Single	Thoracoscopic and laparoscopic	210	Nil	Nil
5	37	Female	2	Segments VI and VII	Single	Nil	170	Nil	Nil
6	52	Male	3	Segments VI, VII, and VIII	Multiple	Laparoscopic to open	250	Nil	Minor bile leak and surgical site infection
7	48	Female	3	Segments VI and VII	Single	Nil	190	Nil	Minor bile leak

The mean operative time was 191.86 minutes. In all the cases, partial pericystectomy with the evacuation of the cyst contents was done. The residual cyst cavity was treated by omentoplasty and tube drainage. One patient in whom the laparoscopic procedure was converted to open procedure developed surgical site infection. Three patients had a minor biliary leak, which improved with conservative management (Table 2).

Patients were discharged on the fourth postoperative day. Removal of port-site sutures was done on the eighth postoperative day at the time of the first review. Patients were followed every month up to three months postoperatively and then every six months for the subsequent three visits for a total of 21 months. All patients underwent USG of the abdomen during follow-up, and there was no evidence of recurrence of the cyst. There was no significant morbidity, mortality, and recurrence at the end of an average 21-month follow-up.

## Discussion

HHC is commonly seen in Asian countries. India is not an exception for any zoonotic diseases, including HHC. Hydatid disease can affect any organ but, the liver is the most common site which gets affected [3]. HHC is the most frequent presentation, ranging from 50% to 93% [4].

Humans get infected either by direct contact with infected dogs or by consumption of unclean fruits and vegetables. In endemic areas, the usual age group of presentation is 30-60 years. However, in non-endemic areas, all age groups are equally affected and more common in the older age group [5]. In our study, all patients were between 30 years and 50 years and were from the non-endemic areas. The incidence of HHC in males and females is equal. However, few studies have reported having male predominance, and few studies have reported female predominance [2,6]. In our study, 70% of the patients were females. 

HHC is a common condition in endemic areas. A most common symptom of HHC is abdominal pain; a similar finding was seen in our study. Others may have abdominal mass, fever, nausea, dyspepsia, or may be asymptomatic. Few patients develop complications due to intraperitoneal or intrabiliary rupture of the cyst, leading to anaphylaxis or sepsis [2].

HHC usually presents as a single cyst; however, it can also be multiloculated or multiple [1,7]. In our study, all patients had a single cyst, and only one patient had multiple cysts. Diagnosis is mainly by imaging and serological testing. Various serological tests like enzyme-linked immunosorbent assay (ELISA), immunoelectrophoresis (IEP) are available, and it reported that the sensitivity of these tests is around 70-90 percent. In all of our patients, we did serological testing with ELISA, and in all the patients, it was positive. USG of the abdomen is effective in the diagnosis of the HHC. Gharbi et al. had classified HHC into five categories based on features on USG (Table 2) [8]. In our study, five had type three, and two had type two cyst. Later, the World Health Organisation (WHO) Informal Working Group on Echinococcosis (IWGE) proposed a modified version of Gharbi classification to identify parasites’ functional states to define the treatment. CECT abdomen is also helpful for diagnosis, which helps to identify the cyst’s exact location in the liver, calcification of cyst wall, infection of the cyst, and impending rupture of the cyst. MRI and endoscopic retrograde cholangiography (ERC) help establish the presence of biliary communication. In our study, all patients underwent CECT, and cyst localization was precisely done. Our patients’ cyst was located in segments VI and VII, and one patient had multiple cysts located in segment VI, VII, and VIII. None of our patients underwent MRI or ERC.

Various modalities of treatment are available. Medical therapy using albendazole alone or in combination with praziquantel is used. Medical treatment is not curative, particularly in an existing HHC. Its primary role is to make the cyst inactive and usually advocated for perioperative management of the disease. Medical therapy with albendazole is also used in disseminated hydatid cyst, patients who are not fit for surgery, peritoneal cyst, and patients with a high risk of recurrence. In our study, all of our patients had received albendazole preoperatively.

Few percutaneous treatment options are available for HHC. These include PAIR and modification like the percutaneous evacuation of cyst content (PEVAC) [9]. However, surgery remains the definitive treatment for HHC. The surgery usually aims at the evacuation of the cyst along with its wall. Open surgical modality is time-tested and is still in practice. The advantages of it are the well-controlled drainage of contents without spillage. Thus the major complication of spillage and anaphylaxis is low. But the access to the lesion needs a large incision. Therefore, it has higher postoperative morbidity in pain, pneumonia, deep vein thrombosis, prolonged hospital stay, and the period to join the work.

There exist various minimally invasive procedures like laparoscopy and robotics. Laparoscopy itself has various modifications. The main fear in laparoscopy was the spillage, inadequate clearance, and anaphylaxis. However, it is documented to be safe, feasible, and beneficial [10,11]. Laparoscopic techniques have their limitations too. These are surgical expertise, availability of advanced laparoscopic equipment/instruments, and patient-related factors. The only non-modifiable factors are patient-related, whereas the rest can be modified, replaced, or upgraded. Among the patient factors, the cyst's location concerning liver segments plays a significant role in completing its laparoscopic cystectomy. The lesions located in the anterior, superior, lateral, and inferior aspects of the liver and superficial ones are easy to deal with laparoscopically. The cysts located on the posterior and posterosuperior aspect of the liver, like Couinaud's segments VI, VII, and VIII, are challenging to deal with laparoscopically and are in the exclusion criteria of many studies [12,13]. Most of the time, these lesions need open access like laparotomy or even thoraco-laparotomy, thus increasing the procedure-related morbidity. In the present study, 70% of the cases in segment VI, VII, and VIII were treated successfully using the laparoscopic closed cystectomy, and 30 percent needed conversion to other modalities like thoraco-laparoscopy and open conversion. We followed the criteria for performing advanced laparoscopic operations established by our previous study in laparoscopic advanced surgery criteria in semi-equipped setup (CLASS) criteria [14].

The main difficulties that arise in the hydatid cyst’s laparoscopic management are accessing the posteriorly located cysts and the evacuation of the particulate contents. There are various methods adopted for the evacuation of the cyst, which include the use of the Palanivelu hydatid system, large transparent beveled cannula, perforator-grinder-aspirator apparatus, liposuction cannula, and perfore-aspirator [2,15-17]. Kayaalp had directly inserted the laparoscopic trocar into the hydatid cyst for the cyst’s evacuation, as similar to our study [18]. In our study, initial decompression of the cyst was done using Veress needle puncture. The cyst was sterilized by instilling the scolicidal agents through the Veress needle. Also, the trocar was placed by the side of the Veress needle. However, Kayaalp reported that their technique is more successful in the anteriorly located cyst and unilocular cyst, in contrast to our study. We completed the closed cystectomy for posteriorly located HHC. Another difficulty in the laparoscopic closed cystectomy management of HHC is the location of the cyst. It can be overcome by choosing appropriate trocar insertion sites and, if needed, by adding additional trocar. Thoracoscopy also can be added if the cyst is located posterosuperior, as we have done in one of our cases. Remnant cyst was dealt with drainage, filling the cavity with normal saline with the closure of cyst wall, introflexion, omentoplasty, marsupialization, and capitonnage. In our study, we managed the remnant cyst in all patients with omentoplasty.

Various reports in the literature suggest that the recurrence rate was 1-22% following open surgery [2]. However, such data for recurrence following laparoscopic surgery is lacking and is not clear. In our study, no patient developed any recurrence during the next 21 months of follow-up. The secret of laparoscopy success for these problematic areas is broad bore access and additional suction cannulas directly into the cyst cavity (with thoracoscopic access if necessary), which avoids spillage and contamination in the operating, table, and early efficient suction drainage of fluid, and daughter cysts. Also, laparoscopy had reduced perioperative blood loss compared to open surgery.

## Conclusions

The laparoscopic closed cystectomy of segments VI, VII, and VIII HHC cyst is feasible, safe, and cost-effective. Thorough preoperative evaluation, preparation, and radiological planning of the procedure should be done. Adequate access with well-positioned ports (with/without thoracoscopy) should be used. Early and wide bore suction decompression of cyst contents should be done. Moreover, finally, experienced laparoscopic surgeons with adequate expertise may be required.
